# Quantitative Measurement of Hoover's Sign: Development of a Standardized Sensor, Interface Procedure, and Testing in Healthy Individuals

**DOI:** 10.1002/brb3.71893

**Published:** 2026-11-03

**Authors:** Takamichi Kanbayashi, Adarsh Nigam, Ciara Pletcher, Alexander White, Srinjoy Mitra, Jon Stone

**Affiliations:** ^1^ Department of Neurology Teikyo University School of Medicine Itabashi‐ku Tokyo Japan; ^2^ Centre for Clinical Brain Sciences University of Edinburgh Edinburgh UK; ^3^ School of Engineering The University of Edinburgh Edinburgh UK

**Keywords:** functional limb weakness, functional neurological disorders, Hoover's sign, quantitative analysis

## Abstract

**Background:**

Hoover's sign is a commonly used clinical sign for diagnosing functional leg weakness, a feature of functional neurological disorder (FND). However, it relies on clinicians’ qualitative assessment. Therefore, more reliable and objective assessment methods are needed. This study aims to develop a portable device and procedure for standardized quantitative measurement of Hoover's sign and validate its utility in healthy controls feigning leg weakness.

**Methods:**

Healthy volunteers without medical backgrounds were recruited. A portable floor‐based device with a pressure sensor placed under the heel of one foot was developed to measure hip extension strength during voluntary and involuntary movements. Subjects performed the test under three conditions: healthy, feigning mild weakness, and feigning severe weakness. The involuntary/voluntary ratio (IV/V ratio) and the side ratio (the IV/V ratio of the feigning leg compared to the non‐feigning leg) were evaluated.

**Results:**

Forty‐two subjects were recruited. In the healthy state, the mean IV/V ratio was 0.60 ± 0.23 on the right side and 0.62 ± 0.24 on the left. The IV/V ratio was significantly higher in the feigned leg during both mild (1.26 ± 0.49) and severe (1.83 ± 0.70) feigning compared to the healthy state (*p* < 0.001 for both), while it was significantly lower in the non‐feigned leg during mild (0.40 ± 0.22) and severe (0.32 ± 0.22) feigning (*p* < 0.001 for both). The side ratio also significantly increased during feigning. ROC analysis revealed optimal IV/V ratio cutoffs of 0.93 for distinguishing mild feigning (sensitivity: 77%, specificity: 93%), and 0.95 for severe feigning (sensitivity: 91%, specificity: 94%).

**Conclusions:**

This portable device can provide a standardized quantitative measure of Hoover's sign and may be useful in future studies of functional leg weakness, particularly in patients with comorbid conditions.

## Graphical abstract

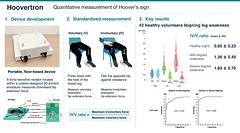

## 1 Introduction

Functional neurological disorder (FND) is one of the commonest clinical conditions in neurological practice and is associated with disability and distress (Gelauff and Stone 2016; Hallett et al. 2022; Stone et al. 2010). Functional limb weakness is one of the major phenotypes of FND and is diagnosed based on bedside examination maneuvers, which highlight a difference between automatic and voluntary movement (American Psychiatric Association 2021; Aybek and Perez 2022).

Hoover's sign is the most commonly used clinical sign for detecting functional leg weakness. First described in 1908, it primarily involves demonstrating the presence of hip extension weakness, which is not present when testing contralateral hip flexion against resistance (Hoover 1908). A second Hoover's sign manoeuver in which the examiner looks for contralateral hip extension during testing of hip flexion in the weak leg is less commonly used. The sign was noted to be present in patients with FND, where leg weakness is experienced as involuntary, but also those feigning or exaggerating weakness. High sensitivity, specificity, and inter‐rater reliability have been reported for Hoover's sign, although it is difficult to carry out such studies blind to the diagnosis or avoid a circularity that the sign itself has been used to make the diagnosis (diagnostic suspicion bias) (Daum et al. 2014; Daum et al. 2015; Mcwhirter et al. 2011; Stone et al. 2010). In addition, assessing the presence or absence of a positive sign, including Hoover's sign, is reported qualitatively by a clinician, typically without defining a standardized technique and requires expertise and training for appropriate use.

Reliable and readily available quantitative methods for evaluating clinical features of FND such as Hoover's sign are a critical unmet need for the field. This is especially the case when investigating FND as a comorbid disorder with other neurological conditions such as multiple sclerosis (Collins et al. 2026; Cuoco et al. 2025; Walzl et al. 2022) or Parkinson's disease (Matar et al. 2024; Wissel et al. 2018). Several past studies have reported quantification of Hoover's sign (Archibald and Wiechec 1970; Arieff et al. 1961; Diukova et al. 2013; Ziv et al. 1998), but the number of evaluated cases is limited, didn't involve a systematic procedure that could be used by a non‐medically trained observer, and is not currently carried out in clinical practice.

In this study, we aimed to develop a portable device for the quantitative evaluation of Hoover's sign that can be used in clinical settings and, as a first step, to conduct pilot testing of its utility in healthy controls moving their legs normally and also feigning mild and severe leg weakness. FND is not feigning (Edwards et al. 2023), but feigning in healthy controls offers an experimental model in which to test quantitative methods of FND diagnosis, since patterns of weakness seen in feigning are similar to that seen in FND (Stone et al. 2017).

## 2 Methods

### 2.1 Subjects

Healthy, non‐medical, volunteers who were acquaintances of the authors were recruited. The inclusion criteria were as follows: (1) no medical history or pain affecting strength in the lower limbs, and (2) do not have specialized knowledge about FND. Exclusion criteria were individuals under 18 years old and those unable to consent to participate in this study. We established which leg was dominant with the question “which foot do you kick a ball with?”

The study design was approved by the South East Scotland Research Ethics Committee (REC reference: 25/SS/0114). Informed consent was obtained from all participants prior to participation.

### 2.2 Development of the Device and Software

We collaborated with engineers (AN, AW, SM) through a process of developing a device and software for the quantitative measurement of hip extension strength. Early iterations involved two devices hand‐held devices with sensors designed to directly copy the way that clinicians do the test, by pressing on the top of one thigh while holding their hand under the other thigh. However, we found that it was hard to gain consistent measurements with this method because measurement depended too heavily on the examiner carrying out multiple procedures. We therefore moved to a floor‐based device, which the subject placed their heel on. To measure the downward force of the leg accurately, the device was designed to support just the heel of the subject, with the pressure sensor placed right at this point. Over several trials, we established a protocol for this novel floor‐based sensor.

The floor‐based device, known as the “Hoovertron,” is made up of a force sensor (FSR01CE, OHMITE) located within a custom‐made 3D‐printed housing shown in Figure 1 with its design diagram. The “Foot Support” ensures that the heel rests on the top of the sensor area placed on the “Footrest.” The force sensor has an active area of 3.97 × 3.97 cm and is covered by a thin cover (of the same size as the sensor) to prevent wear and tear. The length of the device (10 cm) was designed to ensure that the heel was the only major contact point through which downward force can be delivered to the sensor area. A removable “Foot Extender” can be added to support the entire foot for measuring the force exerted during resting position (“baseline,” described further in the next section). Further details of the electronic hardware are shown in Figure S1.

**FIGURE 1:**
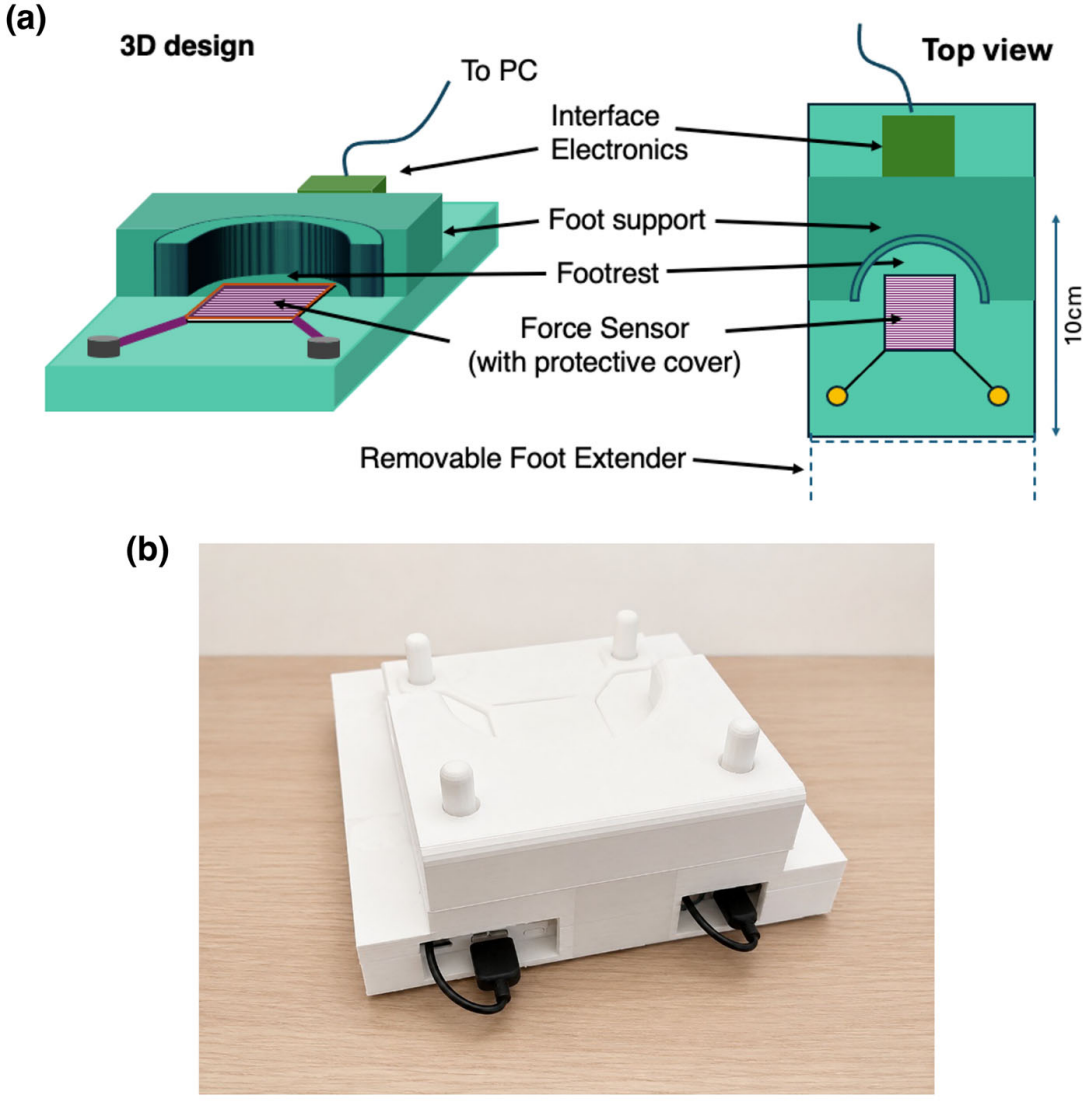
Schematic diagram and photograph of the device. (a) The floor‐based Hoovertron device consists of a force‐sensitive resistor (FSR01CE, OHMITE) housed within a custom‐designed 3D‐printed enclosure. The foot support is designed to position the participant's heel directly over the sensor area, ensuring that downward force generated during hip extension is transmitted to the sensor. (b) A photograph of the device. The background was enhanced using photographic software for presentation purposes.

Data processing to interpret the serial input from the sensor and display data in a user‐friendly way (including CSV reading/writing to save the test and patient information) was implemented python csv and PyQt5.QtSerialPort libraries. The application was designed to enable the extraction of peak force values (measured in Newton) from any range by adjusting the time period if necessary on the measurement screen. Screenshots from the user interface are shown in Figure 2. The details of the Hoovertron concept and design were posted on www.hoovertron.com on 14 Octorber 2026.

**FIGURE 2:**
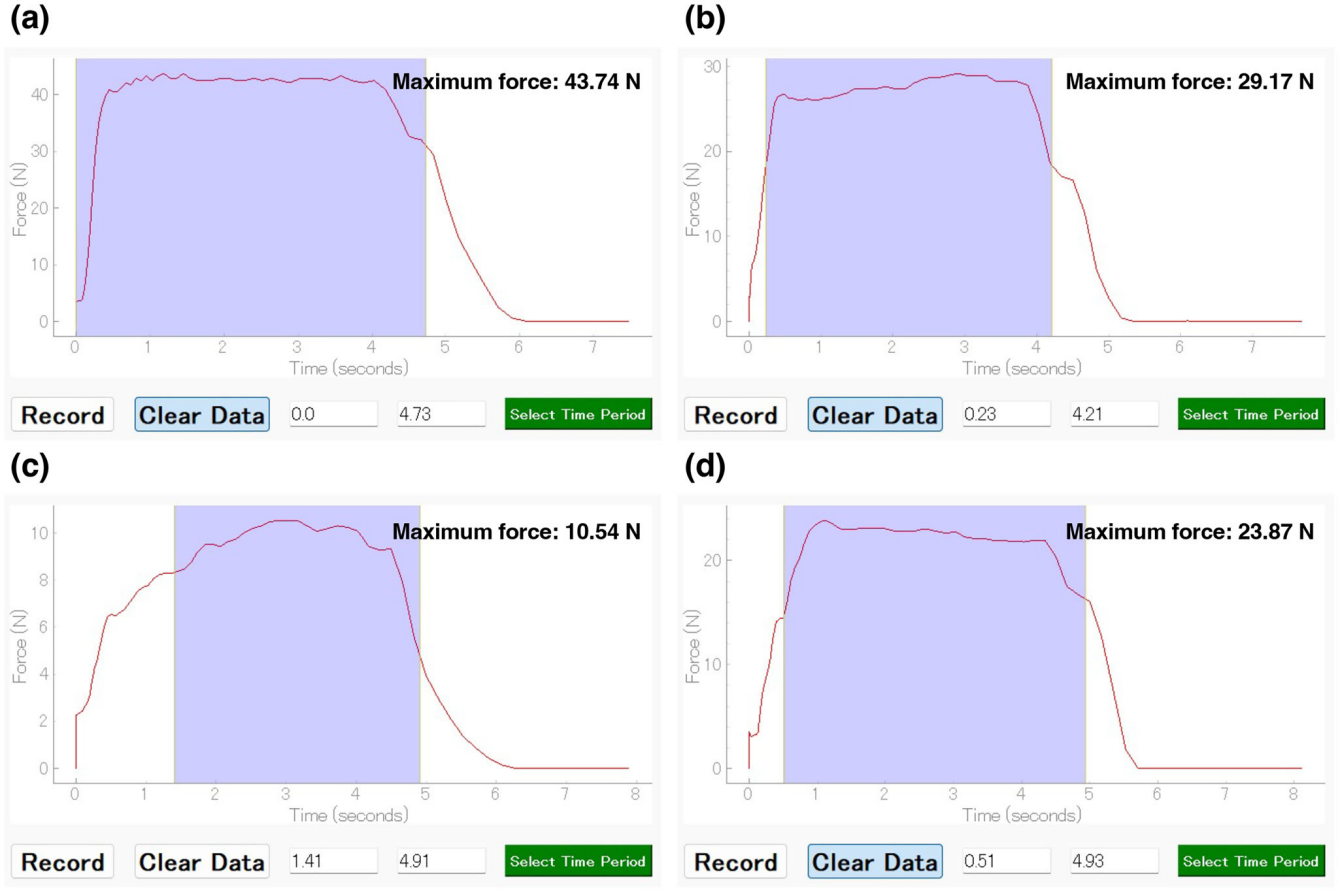
Screenshots of the application's measurement screen in a representative example. Measurements of voluntary (a) and involuntary (b) hip extension force in the healthy condition. Measurements of voluntary (c) and involuntary (d) hip extension force when feigning severe weakness. Force is plotted in Newtons (N) against time (seconds). The light blue rectangle indicates the time span for measurement, which can be adjusted if needed to capture the maximum value, which is the one used for the calculation. The involuntary/voluntary ratio (IV/V ratio), which is defined as the value of the maximum involuntary force divided by the maximum voluntary force, is automatically calculated from each measurement result. In this representative example, the IV/V ratio was 0.67 in the healthy condition and 2.26 when feigning severe weakness. Note the varying value on the *y*‐axis.

### 2.3 Measurement Procedure

We evolved a measurement procedure over time. The measurements were conducted in a seated position using a chair without wheels. All subjects were measured using a standardized measurement procedure with a written protocol including both what the examiner said and how they carried out the test (Supplementary File 1). The test consisted of three parts: (1) a baseline measurement of the weight of the leg. This is not part of a standard Hoover's sign, but it was added to assess the impact of the baseline on the results; (2) voluntary hip extension force; and (3) involuntary hip extension force measurement (Figure 3).

**FIGURE 3:**
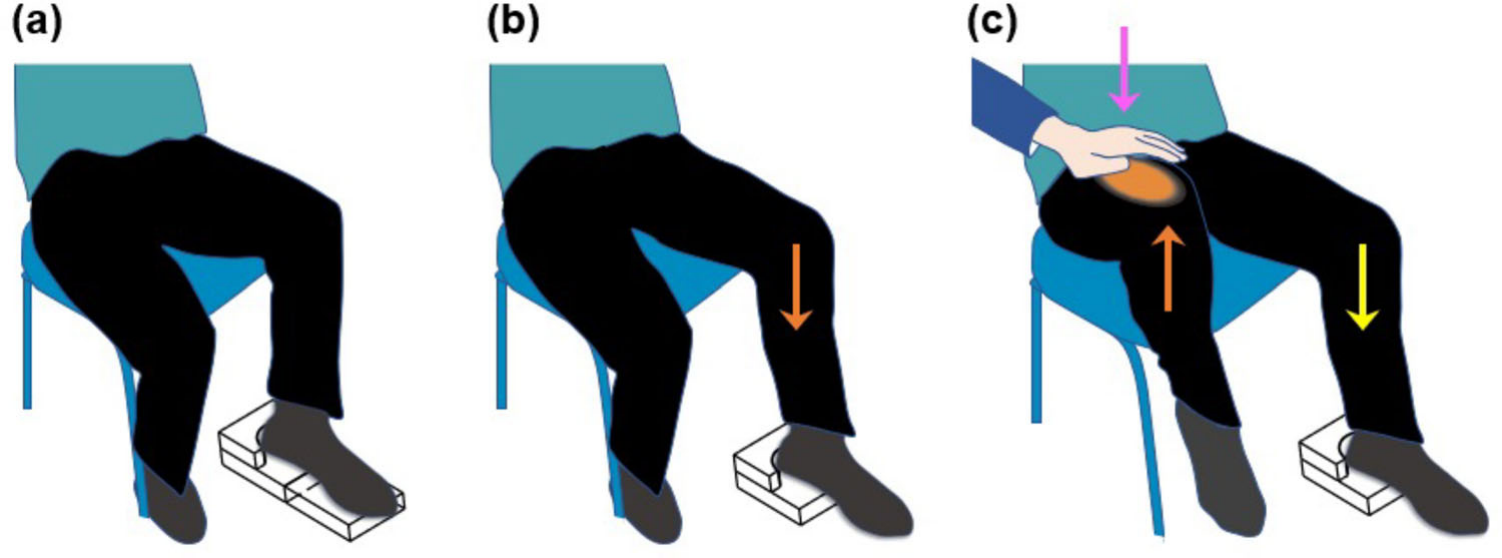
Schematic explanation of the measurement procedures. Baseline measurement (a), voluntary (b), and involuntary (c) force measurements. The orange arrow indicates the direction of the subject's voluntary movement. The yellow arrow indicates the direction of the subject's involuntary movement. The purple arrow indicates the direction of the force applied by the examiner.

During baseline recording, a front part called the “Foot Extender,” which has the same height as the device, was attached to maintain the angle of the ankle, as variations in ankle angle may affect baseline values. Furthermore, subjects were instructed to keep their backs against the chair and relax without applying any downward force with their feet because the baseline values would be influenced by whether the subject was leaning forward or backward.

Second, the front part of the device was removed, and the subject was instructed to press down with one heel of the leg being measured as strongly as possible for at least three seconds to measure the voluntary hip extension downward force. Third, while keeping the same foot on the device, the subject was instructed to focus on the opposite leg and flex their hip with maximum effort against the examiner's “push‐down” force using their hand for at least three seconds, to measure involuntary downward hip extension force. During the voluntary and involuntary force measurement, the posture was unrestricted as we wished to allow patients to perform the test as they would in clinic.

### 2.4 Variation of Task Performance for Study

We performed this procedure as follows: initially, tests were conducted three times on each side for 16 subjects to assess intra‐subject reliability and then on 26 further subjects to reduce volunteer burden, twice on each side:

1. On left and right legs.
2. With the subject instructed to exert force as follows: (1) a healthy condition with no weakness, (2) feign mild weakness, “as if you have weakness but can walk,” and (3) feign severe weakness “severe enough that you cannot walk” in their non‐dominant leg.

### 2.5 Evaluated Parameters

We primarily evaluated the ratio of voluntary to involuntary downward forces, referred to as the “involuntary/voluntary ratio” (IV/V ratio). The IV/V ratio was defined as the value of the maximum involuntary force divided by the maximum voluntary force during measurement.

We also evaluated the “side ratio,” defined as the ratio of the IV/V ratio of the right to the left foot in the healthy state, and the ratio of the IV/V ratio of the feigning leg to the non‐feigning leg in the condition of simulated leg weakness.

The IV/V ratio and side ratio were also calculated and evaluated when the baseline values were subtracted from the measurements of voluntary and involuntary force, respectively.

### 2.6 Statistical Analyses

The intra‐subject reliability of the measurements was evaluated using intra‐class correlation (ICC). The ICC was evaluated only for the IV/V ratio of the dominant foot and the side ratio under healthy conditions. We didn't evaluate the ICC in the feigning condition because the values of the IV/V ratio and side ratio may fluctuate depending on the degree of feigning by the patient. ICC values were interpreted according to the guideline by Portney and Watkins (2000) as follows: excellent (> 0.90), good (0.75–0.89), moderate (0.50–0.74), or poor (< 0.50). The Wilcoxon signed‐rank test was used to evaluate differences in measurement values between the two conditions that were plotted using a violin plot with mean and standard deviation. The Receiver Operating Characteristic (ROC) curve with the minimum distance method was used to determine the optimal cutoff value for distinguishing between healthy and feigned severe or mild weakness states. DeLong's test was applied to compare the area under the curve (AUC) of the two models’ ROC curve. A *p*‐value of < 0.05 was considered statistically significant. The statistical analyses were performed using Excel (Microsoft, Redmond, WA, USA) and R (version 4.2.3, R Foundation for Statistical Computing, Vienna, Austria).

## 3 Results

### 3.1 Enrolled Subjects

A total of 42 healthy volunteers were enrolled in this study, primarily secretaries and non‐medically qualified health professionals working within the hospital. They consisted of 13 males and 29 females, aged from 20 to 62 years (mean ± SD: 38.0 ± 12.0). A total of 39 and 3 subjects had right and left dominant foot, respectively. The baseline value on the right leg was 2.57 ± 1.76 (mean ± SD, range: 0.6–7.3), and that on the left leg was 2.47 ± 1.64 (mean ± SD, range: 0.4–7.9), respectively.

### 3.2 Intra‐Subject Reliability and Effect of Foot Dominance

The ICC for the IV/V ratio based on the data from the first 16 volunteers, who took the test three times each, was 0.8 (with baseline) and 0.8 (without baseline) for the dominant leg, and 0.8 (with baseline) and 0.75 (without baseline) for the non‐dominant leg (all results were within the “good” range). No difference between dominant and non‐dominant legs was found. The ICC for the side ratio was 0.50 (with baseline) and 0.55 (without baseline), both within the “moderate” range. Based on these results, we conducted two tests for each subsequent subject to efficiently collect data from a larger number of participants.

### 3.3 IV/V and Side Ratio in each Situation

The results of the IV/V ratio in each condition are shown in Figure 4. In the following sections, we present only the results *without* baseline subtraction in the manuscript, with those including baseline subtraction provided in Figure S2. This decision was made based on the fact that, as discussed later, excluding the baseline did not significantly influence the results, and good diagnostic performance was observed even without considering the effects of the baseline, which also simplifies the measurement procedure. The figure includes all the data points (in total 100), that is, the three data points from each of the 16 subjects and the 2 data points from each of the remaining subjects.

**FIGURE 4:**
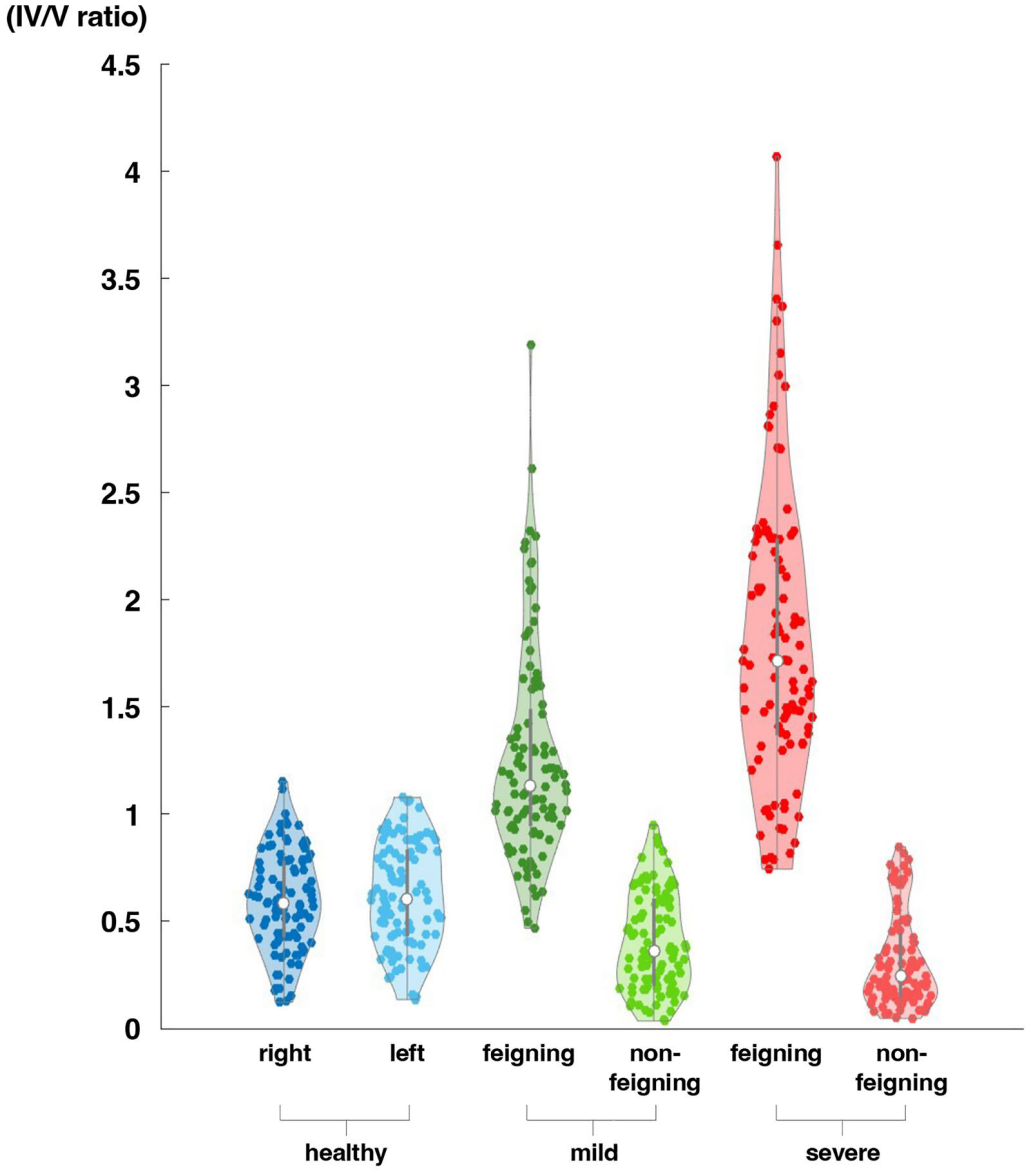
IV/V ratios in the subjects. Violin plots show the distribution of involuntary/voluntary ratios (IV/V ratios). Individual measurements are displayed as dots (100 measurements per condition). White circles indicate mean values and error bars indicate standard deviations. The IV/V ratio in the feigned leg during mild and severe feigned weakness was significantly higher than that in the healthy state (*p* < 0.001 for both). In contrast, the IV/V ratio in the non‐feigned leg during mild and severe feigned weakness was significantly lower than that in the healthy state (*p* < 0.001 for both). Comparisons were performed using the Wilcoxon signed‐rank test.

In healthy volunteers not instructed to feign weakness, the IV/V ratio was 0.60 ± 0.23 (mean ± SD) on the right and 0.62 ± 0.24 on the left, with no statistical difference. Only three subjects had trials with an IV/V ratio exceeding 1. When feigning severe weakness, the IV/V ratio on the feigned leg was 1.83 ± 0.70, and for mild weakness, it was 1.26 ± 0.49. Both values were significantly higher than those in the healthy state (*p* < 0.001 for both). Conversely, the IV/V ratio in the non‐feigned leg was 0.32 ± 0.22 when feigning severe weakness and 0.40 ± 0.22 for mild weakness, both of which were significantly lower than those in the healthy state (*p* < 0.001 for both).

Consequently, the side ratio in the healthy state was 1.03 ± 0.39 and for feigning severe weakness and mild weakness were 10.36 ± 9.98 and 4.91 ± 5.13, respectively (Figure S3). The values of the side ratio for severe or mild feigning were significantly higher than those in the healthy state (*p* < 0.001 for both).

### 3.4 Cutoff Values of IV/V and Side Ratio

ROC curves for the IV/V ratio are shown in Figure 5, and those for the side ratio are presented in Figure S4. The optimal cut‐off value of the IV/V ratio calculated using the ROC curve for the healthy state and mild feigning weakness was 0.93, with sensitivity and specificity of 77% and 93%, respectively. Similarly, the optimal cutoff value for the healthy state and severe feigning weakness was 0.95, with sensitivity and specificity of 91% and 94%, respectively. Consequently, using these optimal cutoff values, out of the 100 data points in the feigned leg, 23 (23%) in mild weakness and 9 (9%) in severe weakness were below the cutoff values, respectively.

**FIGURE 5:**
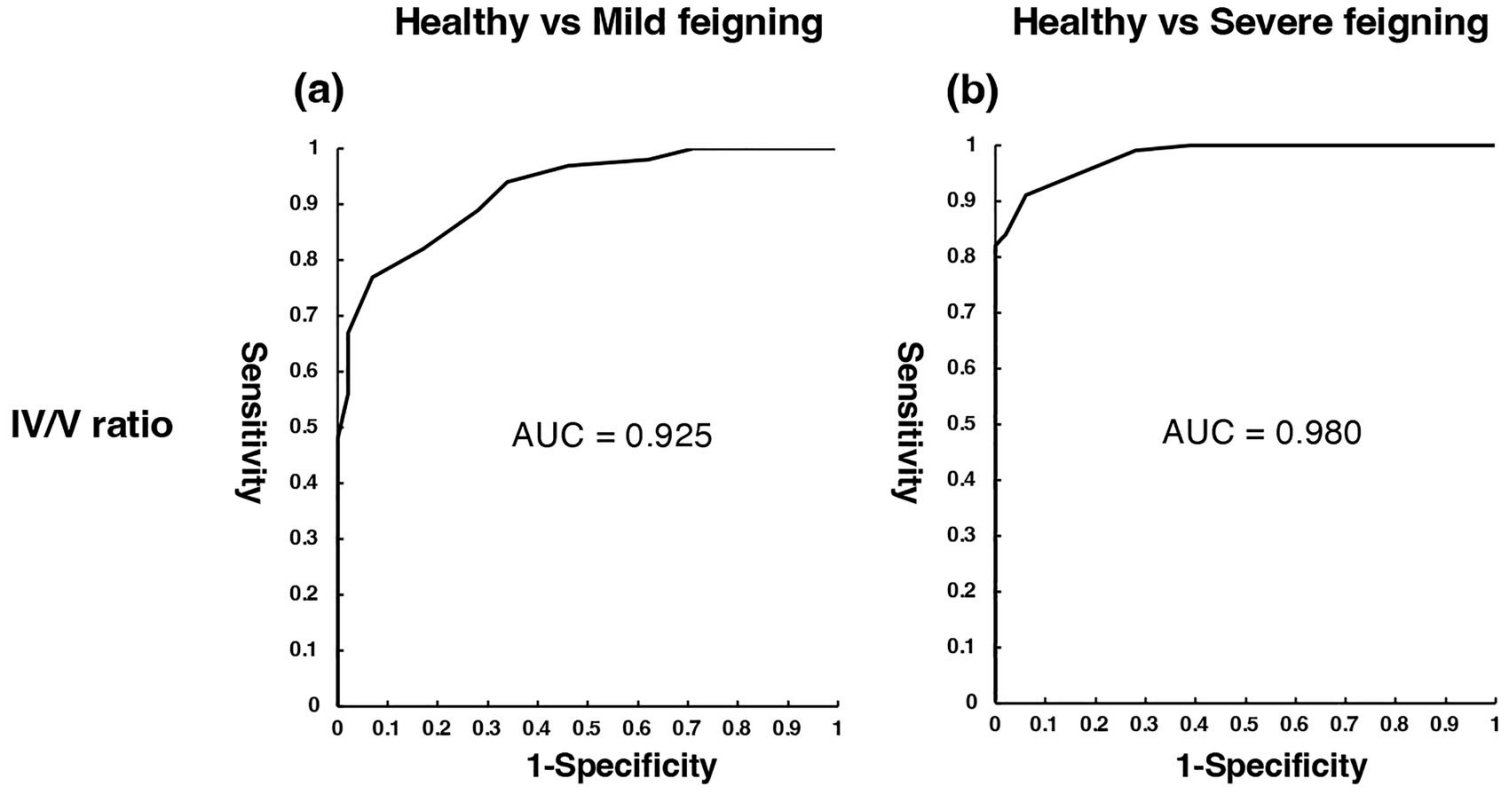
Receiver Operating Characteristic (ROC) curves for determining optimal cut‐off values for the IV/V ratio. (a) ROC curve for discrimination between the healthy state and mild feigned leg weakness. The AUC was 0.925. The optimal cut‐off value was 0.93, with a sensitivity of 77% and specificity of 93%. (b) ROC curve for discrimination between the healthy state and severe feigned leg weakness. The AUC was 0.980. The optimal cut‐off value was 0.95, with a sensitivity of 91% and specificity of 94%. Optimal cut‐off values were determined using the minimum‐distance method.

The optimal cut‐off value of the side ratio for the healthy state and mild feigning weakness was 1.50, with sensitivity and specificity of 90% and 91%, respectively. Similarly, the cutoff value for the healthy state and severe feigning weakness was 1.70, with sensitivity and specificity of 95% and 92%, respectively.

### 3.5 Comparison of the ROC Curves With and Without Baseline

The ROC curves for the IV/V and side ratios with baseline elimination are shown in Figure S5. No significant difference was found between the ROC curves for the IV/V and side ratios in the healthy state and severe feigning weakness (*p* = 0.09 and 0.26, respectively). On the other hand, for the IV/V and side ratios in the healthy state and mild feigning weakness, the ROC curves showed that the AUC was significantly larger when the baseline was not removed (*p* < 0.05 for both).

## 4 Discussion

In this study, we developed a readily portable device for quantitative and standardized measurement of Hoover's sign. Additionally, we demonstrated that this device has the potential to discriminate between healthy individuals and healthy individuals feigning mild and severe leg weakness with high sensitivity and specificity. Functional leg weakness is not feigning (Edwards et al. 2023) but feigned weakness offers an experimental model with similarities to functional leg weakness based on experience with healthy volunteers (Stone et al. 2017).

One of the challenges in FND research is the need for standardized and more objective diagnostic criteria to improve the precision of the diagnosis. For example, there have been studies that have applied objective electrophysiological findings as laboratory‐supported diagnostic criteria for functional tremor (Schwingenschuh et al. 2011; Schwingenschuh et al. 2016). In a similar way, the device we developed also has the potential to provide an objective and standardized quantitative assessment of Hoover's sign, which may be useful in future studies of functional leg weakness.

There are two studies in the last 30 years that described quantitative measurement of Hoover's sign, that is, the voluntary and involuntary force of hip extension, and aimed to establish cut‐off values for discriminating between functional leg weakness and other conditions. One used a strain gauge and a computerized quantitative method (Ziv et al. 1998), and another one used a weighing scale (Diukova et al. 2013); both were conducted in the supine position. Their results showed that their quantitative assessment method clearly distinguished between healthy individuals or patients with leg weakness due to structural abnormalities, and patients with functional leg weakness. However, both studies were based on data from 10 or less subjects, and neither has been applied in clinical practice to date. In the present study, with the expectation of future clinical applications, we developed a device that can be used in clinical settings by a non‐medically trained observer, including outpatient clinics and hospital wards, and obtained data from a larger number of healthy subjects compared to past studies.

Hoover's sign is considered to rely on synergistic contraction (Pearce 2003). Specifically, involuntary hip extension typically occurs during maximal effort to flex the contralateral hip. If an equal force of hip extension were generated on the contralateral side in response to ipsilateral hip flexion, the IV/V ratio should approach 1. Although some individuals showed a ratio exceeding 1, our findings suggest that the force of contralateral hip extension during ipsilateral hip flexion is approximately 60% of that generated voluntarily in most individuals. The results may also be influenced by factors such as the posture during the test (we conducted the test in a seated position); however, this trend was consistent with the results of previous similar studies (Diukova et al. 2013; Ziv et al. 1998).

Our results showed that the more severe the feigned leg weakness, the higher the IV/V and side ratios were compared to the healthy state. In a previous study, we showed that among healthy subjects who pretend to have unilateral leg weakness, more than 20% were able to maintain hip extension weakness while maximally performing contralateral hip flexion (Portney and Watkins 2000). This may account for some of the false positive results we obtained. Clinical experience suggests that this ability to maintain hip extension weakness is much less common in individuals with FND. This raises the possibility that the device may perform better in actual patients with FND, although this requires confirmation in future studies.

The results of this study also confirm data from our previous study, but in a larger group of people, that when asked to feign weakness, healthy controls use an “idea” of what leg weakness is, which is similar to that seen in FND and different to that seen in other causes of limb weakness (e.g., people with multiple sclerosis and stroke, unless densely hemiplegic, usually have good hip extension strength). Hoover's sign can be seen primarily as evidence of impaired agency of a limb, rather than weakness of a limb, which is why it can be false positive in conditions where there is parietal lobe damage such as apraxia (Ercoli and Stone 2020), but also in feigning.

In this study, the IV/V ratios in the non‐feigned leg during the feigned conditions were significantly lower compared to the healthy state. These results suggest that in healthy subjects without neurological structural problems, when attempting maximal hip flexion in one leg but being unable to perform that movement (similar to patients with functional leg weakness), the involuntary hip extension movement of the contralateral leg also becomes weakened. This phenomenon can be considered to represent the complementary aspect of Hoover's sign. Hoover described this phenomenon as the lack of complemental opposition from the normal leg and noted that it is also observed in individuals with functional hemiplegia (Hoover 1908).

The device we developed objectively records the force of hip extension, which is associated with Hoover's sign. However, there are other positive signs that are also useful for diagnosing functional leg weakness, such as the hip abductor sign (Sonoo 2004) and weak gluteus maximus (Sonoo et al. 2023). The diagnosis of FND, including functional limb weakness, should ordinarily be based on multiple positive signs.

If, however, quantitative, objective assessment of other positive signs can be achieved, it would contribute to a more accurate diagnosis of functional limb weakness. Studies in a range of common neurological conditions including multiple sclerosis (Collins et al. 2026; Cuoco et al. 2025; Walzl et al. 2022), Parkinson's disease (Matar et al. 2024; Wissel et al. 2018), and stroke find that around 10%–15% of individuals have comorbid functional neurological symptoms (Stone et al. 2012), with leg weakness being one of the most frequent. This is important since functional leg weakness can be seen as a comorbidity, like pain or depression, which has a specific evidence‐based treatment including FND‐focused physiotherapy. However, without a radiological or laboratory marker it is difficult studying comorbid FND, which is why quantification of the clinical signs used to make the diagnosis will be important.

There are some limitations to this study. First, we did not measure inter‐observer variability in this initial study or whether data changes with additional observer training, for example by video. Second, we evaluated the IV/V and side ratios in healthy subjects feigning unilateral weakness. Although feigning in healthy volunteers provides a useful experimental model for preliminary testing of quantitative measurement procedures relevant to functional leg weakness, it is not the same as testing someone with a clinical disorder. We therefore don't know whether individuals with functional leg weakness will demonstrate similar data, with higher or lower sensitivity than these healthy controls. Furthermore, ratio‐based measures may be influenced by denominator effects when voluntary force becomes very small. Additionally, as is the case with the Hoover's sign itself, this method cannot be applied to patients who are unable to flex their hips. In a real clinical setting, other issues such as pain or fatigue are likely to impact on performance. Furthermore, the measurement procedure remains partially examiner‐dependent, and biomechanical factors such as posture and compensatory strategies may influence performance. Formal engineering validation of the device, including assessment of calibration accuracy, repeatability, drift, hysteresis, heel‐placement variability, and usability in routine clinical settings, was beyond the scope of this initial study and will require future investigation. Finally, repeated measurements from the same participants were included in the analysis and therefore cannot be considered fully independent observations. The ROC‐derived cut‐off values should be regarded as preliminary and require validation in independent clinical cohorts.

Despite these limitations, our device is portable and can be used anywhere, including at the bedside, and has the potential to be applied as a laboratory‐supported tool for diagnosing functional leg weakness in clinical practice and research settings.

## Author Contributions

**Alexander White**: software, data curation, investigation. **Takamichi Kanbayashi**: conceptualization, methodology, data curation, investigation, validation, formal analysis, visualization, writing – original draft. **Srinjoy Mitra**: conceptualization, methodology, software, funding acquisition, project administration, resources, writing – review and editing, investigation, data curation. **Jon Stone**: conceptualization, methodology, validation, supervision, funding acquisition, visualization, project administration, resources, writing – review and editing. **Adarsh Nigam**: software, data curation, investigation. **Ciara Pletcher**: software, data curation, investigation.

## Funding

The study received funding support by two separate awards from NHS Lothian Charities, a Wellcome Institutional Translational Partnership (iTPA) from the University of Edinburgh and a contribution from the FND research fund at the University of Edinburgh, as well as from the University of Edinburgh's “Harmonised Impact Acceleration H020 (IRM Ref: RE16848).”

## Ethics Statement

This study was conducted in accordance with the Declaration of Helsinki and approved by the South East Scotland Research Ethics Committee (REC reference: 25/SS/0114).

## Conflicts of Interest

The authors declare no conflicts of interest. J. Stone has received publishing royalties from UpToDate and carries out expert witness medicolegal work in the United Kingdom. He is president of the FND society and runs a free website neurosymptoms.org for people with FND.

## Supporting information




**Supplementary file 1**. Written protocol for the Standardized Measurement procedure.
Before the test
• Ensure that the PC monitor used for measurements is not visible to the subject during the test.
• Confirm the subject's name, age, sex, and dominant foot (ask which side they use to kick the ball).
• The subject undergoes the test in a seated position (prepare a chair without wheels that can be fixed in place).
• Place the foot (no shoes) of the tested side onto the device and fit the heel onto the device (e.g., “Please make sure your heel is securely fitted on the device”).
**1. Baseline measurement**:
Device: attach a part of the same height to the front of the device to keep the foot flat.
Posture: keep the subject's back against the chair and have them clasp their hands behind it.
• Just placing the foot on the device without any voluntary downward force.
• Record the baseline values both right and left.
**2. Voluntary measurement**:
Device: the front part of the device was removed.
Posture: no limitation.
• The examiner explains to the subject how they should do (e.g., “Push down with your heel as hard as you can for 3 s”).
• After stopping the recording, adjust the time span on the monitor to obtain the value of maximal force.
**3. Involuntary measurement**:
Device: the front part of the device was removed.
Posture: no limitation.
• The examiner places their hand on the knee of the non‐tested leg and explains to the subject how they should do (e.g., “Lift up your knee and stop me from pressing down as hard as you can for 3 s”).
*Note 1: At this time, ensure that the tested‐side foot remains on the device and direct the subject's attention to the non‐tested leg.
*Note 2: Apply downward resistance after confirming that the leg is elevated. During the test with feigning, most subjects may not be able to elevate the weak leg; however, at least when instructing the elevation of the good leg, please ensure that it is elevated before applying downward resistance.
• After stopping the recording, adjust the time span on the monitor.

**Figure S1. Details of electronic hardware**.
The sensor interface electronics are designed using commercial off‐the‐shelf components and implemented on a Printed Circuit Board. It includes an Arduino Nano Every connected with MCP6002 op‐amp. The force sensor (FSR01CE) is used in a voltage divider configuration with a 180 Ω resistor. Further, a 0.1 µF capacitor is connected in parallel with the resistor for stability. The MCP6002 op‐amp is connected as a voltage follower to avoid the loading effect, and the output of the op‐amp is connected to the A0 pin (analog input) pin of Arduino. The complete sensing interface is powered by a +5 V DC supply through the Arduino Nano Every board. The input voltage is converted into a force value in Newtons using a calibration curve derived using a precision force sensor and fitted to a binomial curve. Additionally, a finite impulse response (FIR) filter was applied to one of the sensor signals within the firmware to provide a filtered output. This digital low‐pass filter is necessary to provide an average output. The sampling rate of the force signal is 1 kHz and the low‐pass filter has a cutoff of 10 Hz. The firmware communicates the original and the average data values to the PC through a serial port.

**Figure S2. IV/V and side ratios in the subjects (baseline subtracted)**.
Distribution of IV/V ratios (a) and side ratios (b) after baseline subtraction in the healthy condition and during mild and severe feigned unilateral leg weakness. Individual measurements are displayed as dots (100 measurements per condition). White circles indicate mean values and error bars indicate standard deviations. The IV/V ratio in the feigned leg during mild and severe feigned weakness was significantly higher than in the healthy state (*p* < 0.001 for both). In contrast, the IV/V ratio in the non‐feigned leg during mild and severe feigned weakness was significantly lower compared to the healthy state (*p* < 0.001 for both). The side ratios in mild or severe feigned weakness were significantly higher compared to the healthy state (*p* < 0.001 for both). Comparisons were performed using the Wilcoxon signed‐rank test.

**Figure S3. Side ratios in the subjects (baseline not subtracted)**.
Violin plots show the distribution of side ratios. Individual measurements are displayed as dots (100 measurements per condition). White circles indicate mean values and error bars indicate standard deviations. The side ratio was 1.03 ± 0.39 (mean ± SD) in the healthy state, and 4.91 ± 5.13 and 10.36 ± 9.98 during mild and severe feigned weakness, respectively. The side ratios in mild or severe feigned weakness were significantly higher compared to the healthy state (*p* < 0.001 for both).

**Figure S4. Receiver Operating Characteristic (ROC) curves for determining optimal cut‐off values for the side ratio**.
(a) ROC curve for discrimination between the healthy state and mild feigned leg weakness. The AUC was 0.946. The optimal cut‐off value was 1.50, with a sensitivity of 90% and specificity of 91%. (b) ROC curve for discrimination between the healthy state and severe feigned leg weakness. The AUC was 0.981. The optimal cut‐off value was 1.70, with a sensitivity of 95% and specificity of 92%. Optimal cut‐off values were determined using the minimum‐distance method.

**Figure S5. Receiver Operating Characteristic (ROC) curves for determining optimal cut‐off values (baseline subtracted)**.
(a) ROC curve of the IV/V ratio for discrimination between the healthy state and mild feigned leg weakness. The AUC was 0.913. The optimal cut‐off value was 0.85, with a sensitivity of 81% and specificity of 85%. (b) ROC curve of the IV/V ratio for discrimination between the healthy state and severe feigned leg weakness. The AUC was 0.972. The optimal cut‐off value was 0.95, with a sensitivity of 91% and specificity of 96%. (c) ROC curve of the side ratio for discrimination between the healthy state and mild feigned leg weakness. The AUC was 0.931. The optimal cut‐off value was 1.5, with a sensitivity of 91% and specificity of 88%. (d) ROC curve of the side ratio for discrimination between the healthy state and severe feigned leg weakness. The AUC was 0.977. The optimal cut‐off value was 1.7, with a sensitivity of 97% and specificity of 91%. Optimal cut‐off values were determined using the minimum‐distance method.

## Data Availability

The data that support the findings of this study are available from the corresponding author upon reasonable request.
