# Supplementary material for: Quantitative Measurement of Hoover's Sign: Development of a Standardized Sensor, Interface Procedure, and Testing in Healthy Individuals
Source: Brain Behav. 2026 Nov 3;16(11):e71893. doi: 10.1002/brb3.71893 (PMC13729825; doi:10.1002/brb3.71893)
Supplement: Supplementary file 1 [file BRB3-16-e71893-s001.docx]

**Supplementary file 1.** Written protocol for the Standardized Measurement procedure.

**Before the test**

• Ensure that the PC monitor used for measurements is not visible to the subject during the test.

• Confirm the subject’s name, age, sex, and dominant foot (ask which side they use to kick the ball).

• The subject undergoes the test in a seated position (prepare a chair without wheels that can be fixed in place).

• Place the foot (no shoes) of the tested side onto the device and fit the heel onto the device (e.g., “Please make sure your heel is securely fitted on the device”).

**1. Baseline measurement:**

Device: attach a part of the same height to the front of the device to keep the foot flat.

Posture: keep the subject’s back against the chair and have them clasp their hands behind it.

• Just placing the foot on the device without any voluntary downward force.

• Record the baseline values both right and left.

**2. Voluntary measurement:**

Device: the front part of the device was removed.

Posture: no limitation.

• The examiner explains to the subject how they should do (e.g., “Push down with your heel as hard as you can for 3 seconds”).

• After stopping the recording, adjust the time span on the monitor to obtain the value of maximal force.

**3. Involuntary measurement:**

Device: the front part of the device was removed.

Posture: no limitation.

• The examiner places their hand on the knee of the non-tested leg and explains to the subject how they should do (e.g., “Lift up your knee and stop me from pressing down as hard as you can for 3 seconds”).

*Note1: At this time, ensure that the tested-side foot remains on the device and direct the subject’s attention to the non-tested leg.

*Note2: Apply downward resistance after confirming that the leg is elevated. During the test with feigning, most subjects may not be able to elevate the weak leg; however, at least when instructing the elevation of the good leg, please ensure that it is elevated before applying downward resistance.

• After stopping the recording, adjust the time span on the monitor.
